# Can we predict sleep health based on brain features? A large-scale machine learning study using the UK Biobank

**DOI:** 10.1093/braincomms/fcag016

**Published:** 2026-01-22

**Authors:** Federico Raimondo, Hanwen Bi, Vera Komeyer, Jan Kasper, Sabrina Primus, Felix Hoffstaedter, Synchon Mandal, Laura Waite, Juliane Winkelmann, Konrad Oexle, Simon B Eickhoff, Masoud Tahmasian, Kaustubh R Patil

**Affiliations:** Brain and Behavior (iNM-7), Institute of Neuroscience and Medicine, 52428 Jülich, Germany; Institute for Systems Neuroscience, Medical Faculty, Heinrich-Heine University Düsseldorf, 40225 Düsseldorf, Germany; Brain and Behavior (iNM-7), Institute of Neuroscience and Medicine, 52428 Jülich, Germany; Institute for Systems Neuroscience, Medical Faculty, Heinrich-Heine University Düsseldorf, 40225 Düsseldorf, Germany; Brain and Behavior (iNM-7), Institute of Neuroscience and Medicine, 52428 Jülich, Germany; Institute for Systems Neuroscience, Medical Faculty, Heinrich-Heine University Düsseldorf, 40225 Düsseldorf, Germany; Department of Biology, Faculty of Mathematics and Natural Sciences, Heinrich Heine University Düsseldorf, 40225 Düsseldorf, Germany; Brain and Behavior (iNM-7), Institute of Neuroscience and Medicine, 52428 Jülich, Germany; Institute for Systems Neuroscience, Medical Faculty, Heinrich-Heine University Düsseldorf, 40225 Düsseldorf, Germany; Institute of Neurogenomics (iNG), Helmholtz Zentrum München, D-85764 Munich, Germany; Institute of Human Genetics, TUM School of Medicine and Health, Technical University of Munich, Munich 81675, Germany; Brain and Behavior (iNM-7), Institute of Neuroscience and Medicine, 52428 Jülich, Germany; Institute for Systems Neuroscience, Medical Faculty, Heinrich-Heine University Düsseldorf, 40225 Düsseldorf, Germany; Brain and Behavior (iNM-7), Institute of Neuroscience and Medicine, 52428 Jülich, Germany; Institute for Systems Neuroscience, Medical Faculty, Heinrich-Heine University Düsseldorf, 40225 Düsseldorf, Germany; Brain and Behavior (iNM-7), Institute of Neuroscience and Medicine, 52428 Jülich, Germany; Institute of Neurogenomics (iNG), Helmholtz Zentrum München, D-85764 Munich, Germany; Institute of Human Genetics, TUM School of Medicine and Health, Technical University of Munich, Munich 81675, Germany; Institute of Neurogenomics (iNG), Helmholtz Zentrum München, D-85764 Munich, Germany; Institute of Human Genetics, TUM School of Medicine and Health, Technical University of Munich, Munich 81675, Germany; Brain and Behavior (iNM-7), Institute of Neuroscience and Medicine, 52428 Jülich, Germany; Institute for Systems Neuroscience, Medical Faculty, Heinrich-Heine University Düsseldorf, 40225 Düsseldorf, Germany; Brain and Behavior (iNM-7), Institute of Neuroscience and Medicine, 52428 Jülich, Germany; Institute for Systems Neuroscience, Medical Faculty, Heinrich-Heine University Düsseldorf, 40225 Düsseldorf, Germany; Department of Nuclear Medicine, Faculty of Medicine and University Hospital Cologne, University of Cologne, 50937 Cologne, Germany; Brain and Behavior (iNM-7), Institute of Neuroscience and Medicine, 52428 Jülich, Germany; Institute for Systems Neuroscience, Medical Faculty, Heinrich-Heine University Düsseldorf, 40225 Düsseldorf, Germany

**Keywords:** sleep health, structural MRI, functional MRI, UK Biobank, machine learning

## Abstract

Numerous correlational and group comparison studies have demonstrated robust associations between sleep health (SH) and large-scale brain organization. However, individual differences play a critical role in this relationship, highlighting the need for person-specific analyses. In this study, we aimed to explore whether multiple brain imaging features could predict various SH-related traits at the individual level using machine learning (ML) techniques. We utilized data from 28 088 participants in the UK Biobank, extracting 4677 structural and functional neuroimaging markers. These features were then used to predict a range of self-reported sleep characteristics, including insomnia symptoms, sleep duration, ease of waking in the morning, chronotype, napping behaviour, daytime sleepiness and snoring. For each of these seven traits, we trained both linear and nonlinear ML models to evaluate how well brain imaging data could account for individual differences. Our analyses involved extensive computational resources, equivalent to over 200 000 core-hours (equivalent to 25 years of compute time). Despite this, the predictive performance of brain features was consistently low across all models, with balanced accuracy scores ranging from 0.50 to 0.59. The highest accuracy achieved (0.59) came from a linear model predicting the ease of getting up in the morning. Notably, models using only demographic variables such as age and sex achieved comparable performance, suggesting that these basic characteristics may largely explain the observed variability. These findings indicate that, even when using a large, well-powered sample and advanced ML techniques, multi-modal brain imaging features provide limited predictive value for SH at the individual level. This low predictability underscores the complexity of the relationship between self-reported sleep and brain structure/function. It also suggests that other biological, environmental or psychological factors—possibly not captured by current imaging modalities—may play a more substantial role in shaping sleep-related behaviours.

## Teaser

Raimondo *et al*. report that, using multimodal MRI data from 28 088 UK Biobank participants, machine-learning models could not accurately predict individual sleep-health traits. Balanced accuracies did not exceed 0.59, and demographic variables performed similarly. They conclude that brain-imaging features provide limited predictive value, suggesting other biological or environmental factors underlie sleep-health variation.

## Graphical Abstract

**Graphical Abstract:**
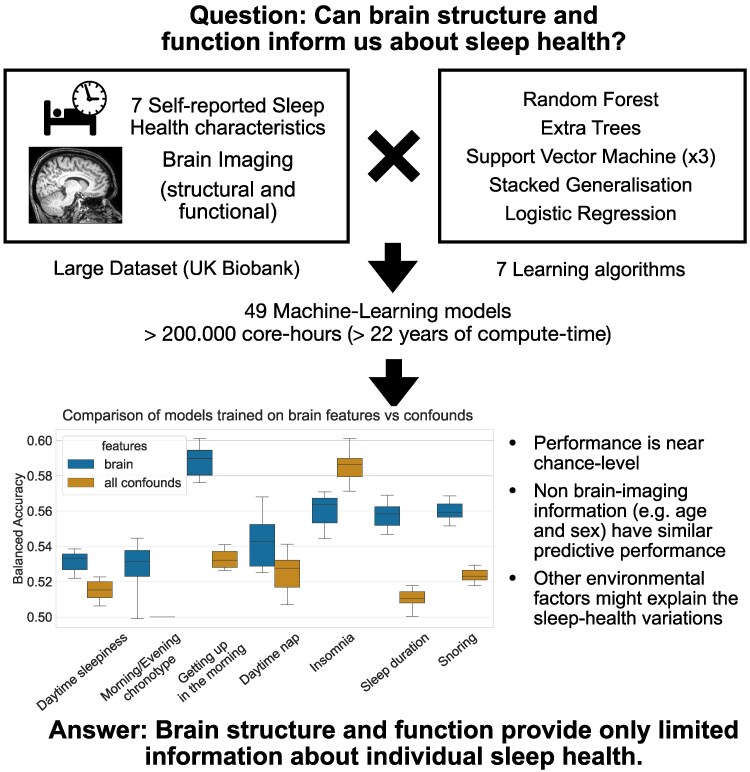

## Introduction

Sleep is a non-negotiable human need that has pivotal impacts on memory processing, metabolite clearance, immune system adaptation, optimal cognition and mental health.^1^ The intricate relationship between sleep health (SH) and brain health has recently garnered significant scientific attention.^2-8^ SH is a multidimensional concept characterized by subjective satisfaction, alertness, regularity, timing and sleep duration,^9^ which is considered a crucial indicator of human well-being. Seven different SH-related characteristics (i.e. sleep duration, easiness/difficulty of getting up in the morning, chronotype, nap, daytime dozing/sleepiness, as well as insomnia symptoms and snoring) reflect various SH dimensions and were collected in half a million participants in the UK Biobank (UKB).^10,11^ This large-scale population data presents a unique opportunity to explore the link between various SH dimensions and brain structure/function, overcoming the low reproducibility of previous small sample studies, as employed previously.^3,5,12-15^

The link between various SH dimensions and brain structure and function has been reported in correlational, group comparison and neuroimaging meta-analysis^16^ studies but pointed to heterogeneous results. Sleep disturbance conditions, including insomnia symptoms,^8,17,18^ sleep-disordered breathing^19-21^ and abnormal sleep duration,^5,22^ demonstrated inconclusive associations between sleep and the brain. For example, (i) for insomnia domain, Schiel *et al*. utilized data from the general population in the UKB, while Weihs *et al*. analysed both the general population and patients with clinical insomnia disorder from the ENIGMA-Sleep datasets. Neither study found a strong association between insomnia symptoms/disorder and grey matter volume (GMV).^8,23^ However, Stolicyn and colleagues using UKB showed that insomnia symptoms are associated with higher global GMV, mainly in the amygdala, hippocampus and putamen.^24^ Moreover, individuals with insomnia symptoms demonstrated altered functional connectivity (FC) within and between the default mode network (DMN), frontoparietal network (FPN), and salience network (SN)^18^; (ii) sleep-disordered breathing and snoring are linked to structural and functional brain alterations and increase the rate of cognitive decline in the general population and patients with dementia^20,21,25^; (iii) regarding sleep duration, one study using UKB data found that short sleep duration is linked with lower amygdala reactivity to a negative facial expression task.^26^ The non-linear associations have been documented between sleep duration, cognitive performance, mental health and a wide range of regional differences in brain structure, mainly in the subcortical areas.^5,23,24,27,28^ Fjell and colleagues performed cross-sectional analyses based on the UKB sample, indicating inverse U-shaped relationships between sleep duration and brain structure, i.e. 6.5 h of sleep was associated with increased cortical thickness and subcortical volumes relative to intracranial volume. However, they failed to identify a longitudinal association between sleep duration and cortical thickness.^4^ In another study, they found that individuals who reported short sleep without other sleep problems or daytime sleepiness had larger brain volumes compared to both short sleepers with sleep issues and daytime sleepiness, as well as those who slept 7–8 h^29^; (iv) analysis of chronotypes also showed that the evening chronotype is linked with higher GMV in the precuneus, bilateral nucleus accumbens, caudate, putamen and thalamus and orbitofrontal cortex.^30^ Robust associations between chronotype and neuroimaging phenotypes, predominantly in the basal ganglia, limbic system, hippocampus and cerebellum have been reported using UKB,^31^ which can be mediated by genetic factors^32^; (v) self-reported daytime sleepiness has been reported to be related to higher cortical GMV^33^; (vi) A Mendelian randomization study in UKB found causal association between genetic liability of daytime napping and larger total brain volume but not hippocampal volume;^34^ (vii) difficulty in getting up in the morning, which can be the symptoms of various SH domains, particularly late chronotype, insomnia and snoring, is also related to brain abnormalities.^24,31^ These findings together represent an overall inconsistency in the relationship between SH domains and the brain. While these studies provided valuable insights, they mostly used case–control or correlational designs and might not have been able to capture the complex linear and non-linear interplay between the brain and SH,^35^ which is a heterogeneous subjective concept that varies across individuals. Thus, the substantial inter-individual variability of SH and the differential associations between various SH characteristics and brain measurements necessitate large-scale datasets and more advanced computational approaches to better model this complexity,^36,37^ to improve our understanding of neurobiological substrates and behavioural consequences of sleep–brain interaction.

Machine learning (ML) offers a powerful tool to unravel complex relationships, providing a more nuanced representation than traditional statistical approaches, which is critical in personalized treatment in sleep medicine.^38,39^ ML models can consider complex multivariate linear and non-linear relations to make brain behaviour predictions on unseen brain imaging data and have the potential to identify generalizable patterns in SH-related neurobiology at the individual subject level,^40-42^ surpassing conventional group comparisons and correlations. In particular, non-linear models are necessary to capture sleep duration–brain interplay. Accurate predictive models can contribute to refining our theoretical understanding of the SH–brain relationship. This might pave the way for developing more effective clinical strategies to enable personalized interventions and treatments.^43^ Directional genetic analyses using Mendelian randomization demonstrated that altered SH dimensions are more a consequence than a cause of brain abnormalities.^44^

Thus, we considered SH characteristics as targets of ML models.

In this work, we leveraged the extensive neuroimaging dataset from the UKB to investigate whether multi-modal brain measures—such as grey matter volume, surface-based morphometry and intrinsic functional metrics (local correlation (LCOR), global correlation (GCOR) and fractional amplitude of low-frequency fluctuations (fALFF))—can reliably distinguish distinct states associated with 7 SH traits. Our goal was to determine if brain imagining, independent of simple demographic variables such as age and sex, can differentiate between well-separated conditions in each SH-related characteristic (e.g. differentiating individuals who are usually having insomnia symptoms from individuals without insomnia symptoms).

## Materials and methods

### Participants

We selected the data of the first imaging visit (instance 2) from the UKB (http://www.ukbiobank.ac.uk), recorded from 2014 onwards at three different sites in the UK (Cheadle, Reading, Newcastle). The acquisition parameters and protocol of both the structural and functional MRI are as described previously.^11^ We included all individuals who participated in the imaging session, and their data had already been pre-processed and denoised by the UKB team.^45^ Thus, no particular in-/exclusion criteria have been applied in this sample to be representative of the general population. We selected the individuals for whom all features were computed, resulting in a total *N* of 28,088, 47% male and 64.1 years old on average (58–78 years IQR) and included them (more demographic variables in Supplementary Table 1). The UKB project is approved by the NHS National Research Ethics Service (Ref. 11/NW/0382), and all participants gave written informed consent before participation. Ethical standards are continuously controlled by an Ethics Advisory Committee (EAC, http://www.ukbiobank.ac.uk/ethics), based on a project-specific Ethics and Governance Framework (https://www.ukbiobank.ac.uk/wp-content/uploads/2025/01/Ethics-and-governance-framework.pdf). The current analyses were conducted under UK Biobank application number 41655. STROBE guidelines for cohort studies were followed in this study.

### Sleep health characteristics

The multifaceted definition of SH in the UKB is based on previous SH studies.^3,9,18,23,26,46^ Accordingly, the seven SH-related characteristics were self-reported insomnia symptoms, sleep duration, difficulty/easiness of getting up in the morning, chronotype, daily nap, daytime sleepiness, and snoring (category 100057), obtained from the touchscreen questionnaire. As these questions were asked at every visit, we selected the responses from the visit matching the neuroimaging acquisition visit. Following is a list of the origin of the SH-related characteristics, including the questions and field IDs from the UKB, for reproducibility purposes.

- Sleeplessness/insomnia field (field 1200): ‘Do you have trouble falling asleep at night or do you wake up in the middle of the night?’, which could be answered as ‘never/rarely’, ‘sometimes’, ‘usually’ or ‘prefer not to answer’.
- Sleep duration (field 1160): ‘How many hours sleep do you get in every 24 h?’.
- Getting up in the morning (field 1170): ‘On average a day, how easy do you find getting up in the morning?’, with four answers spanning from not at all easy to very easy, as well as ‘do not know’ and ‘prefer not to answer’.
- Chronotype (i.e. morning/evening person, field 1180): ‘What do you consider yourself to be?’, with four possible answers spanning from a ‘morning person’ to an ‘evening person’, as well as ‘do not know’ and ‘prefer not to answer’.
- Nap during the day (field 1190): ‘Do you have a nap during the day?’, which can be answered as ‘never/rarely’, ‘sometimes’, ‘usually’ or ‘prefer not to answer’.
- Daytime dozing (field 1220): ‘How likely are you to doze off or fall asleep during the daytime when you don't mean to? (e.g. when working, reading or driving)’, which can be answered as ‘never/rarely’, ‘sometimes’, ‘often’ or ‘prefer not to answer’.
- Snoring (field 1210): ‘Does your partner or a close relative or friend complain about your snoring?’, with ‘yes’, ‘no’, ‘do not know’ and ‘prefer not to answer’ as possible answers.

Given the ambiguous meaning that some questions, and consequently the respective answers, potentially have in the UKB data (e.g. ‘sometimes’ versus ‘often’), and to simplify the multiclass/continuous target problems into binary classification problems, we first analysed the performance of models aimed at distinguishing the extreme answers of each SH-related characteristic. In the case of the continuous answer regarding sleep duration in hours, we split the distribution into four quantiles, selecting the first and fourth quantiles as two classes. However, given the concentration of answers around the median (7 h), this resulted in discarding only the samples that replied 7 h. The rationale behind considering the extreme values as class labels is to simplify the classification task, resulting in higher predictive performance if there is indeed a relationship between brain imaging data and each SH-related characteristic. A description of the considered answers for each question, as well as the number of samples for each class, can be seen in Table 1.

**Table 1:** List of answers used for each SH-related characteristic to convert the ambiguous answers into binary classification problems

Sleep health-related characteristic	Extreme values			
	Class 0		Class 1	
	Answer(s)	#Samples	Answer(s)	#Samples
**Insomnia symptoms**	‘Never/rarely’	6127	‘Usually’	8846
Sleep duration	1st quantile [0–6]	6760	4th quantile [8–16]	9959
Getting up in the morning	‘Very Easy’	10687	‘Not at all easy’ ‘Not very easy’	3554
Morning/Evening chronotype	‘Definitely a ‘morning’ person’	7145	‘Definitely an ‘evening’ person’	2398
Daytime nap	‘Never/rarely’	15915	‘Usually’	1565
Daytime sleepiness **^a^**	‘Never/rarely’	21406	‘Sometimes’ ‘Often’	6458
Snoring **^a^**	‘Yes’	16439	‘No’	9469

^a^Denotes the questions for which no samples were dropped.

### Processing of imaging data

#### Structural imaging (T1)

Grey Matter Volume (GMV): T1-weighted pre-processed images were retrieved from UKB with subsequent computations of voxel-based morphometry (CAT 12.7 (default settings); MNI152 space; 1.5 mm isotropic).^47^

Brain Surface: We used the T1-weighted data processed using FreeSurfer 6.0 as provided by the UKB (see https://git.fmrib.ox.ac.uk/falmagro/UK_biobank_pipeline_v_1/-/tree/master/bb_FS_pipeline for the exact pipeline used). This includes grey/white matter contrast, pial surface, white matter surface, white matter thickness and white matter volume from the 68 ROIs of the Desikan-Kiliany parcellation,^48^ totalling 328 features.

#### Functional imaging (fMRI)

Resting-state Functional Magnetic Resonance Imaging (rsfMRI): The fractional amplitude of low-frequency fluctuations (fALFF) represents the relative measure of blood oxygenation level-dependent (BOLD) magnetic resonance signal power within the low-frequency band of interest (0.008–0.09 Hz, reflecting the spontaneous neural activity of the brain) as compared to the BOLD signal power over the entire frequency spectrum.^49^ The LCOR (‘local correlation’) is a metric that represents the local coherence for each voxel. It is computed as the average of correlation coefficients between a voxel and a region of neighbouring voxels, defined by a 25 mm Gaussian kernel.^50^ On the other hand, the GCOR (‘global correlation’) represents the node centrality of each voxel and is computed as the average of the correlation coefficients between a voxel and all voxels of the whole brain. These metrics were calculated using MatLab2020b, SPM12,^51^ FSL (version 5.0)^52^ and the CONN toolbox.^53^

#### Feature extraction

The voxel-wise data from VBM and fMRI data were then aggregated employing the voxel-wise winsorized mean (10% limits) for each region of interest (ROI) of the cortical Schaefer atlas (1000 ROIs),^54^ the Melbourne subcortical atlas (S4 3T, 54 ROIs)^55^ and the Diedrichsen cerebellar atlas (SUIT space, 34 ROIs).^56^ This resulted in 1088 GMV features extracted and 1087 features for each fMRI-derived metric (fALFF, LCOR and GCOR). Note that Diedrichsen cerebellar atlas produced 33 features for the fMRI data as for some ROIs, there were not enough voxels to compute the values correctly. The number of variables and samples for each neuroimaging feature is described in Supplementary Table 2.

### ML models

**Figure 1:**
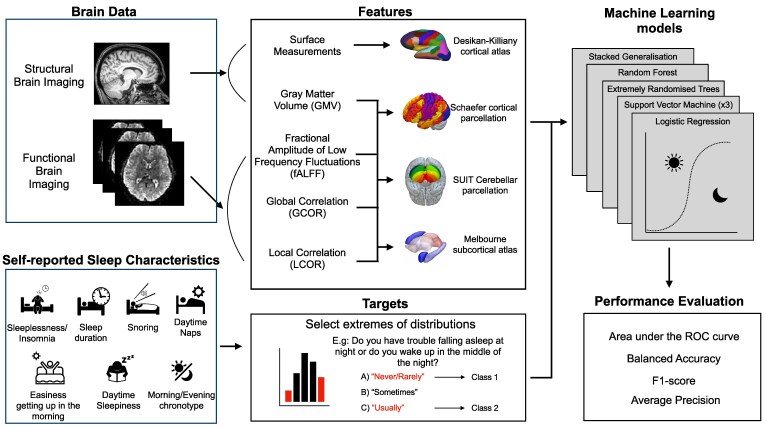
**Overview of the methodology.** The brain images were processed in order to obtain cortical and subcortical features, both from structural and functional brain imaging. Answers for the UK Biobank questionnaire were binarized by selecting the extremes of the distributions as described in Table 1. We then evaluated the out-of-sample performance of 7 different ML models, independently for each SH-related characteristic.

In order to evaluate a broad spectrum of possible interactions between features and relations to the targets, we selected five ML algorithms, including parametric and non-parametric models, testing for linear and nonlinear relations. We tested a Random Forest,^57^ extremely randomized trees (Extra Trees),^58^ support vector machine (SVM),^59^ logistic regression (logit) and stacked generalization,^60^ with different hyperparameter settings, resulting in seven models. Table 2 summarizes the models, including the hyperparameters tested, except for the stacked generalization model, which is described below. When more than one hyperparameter value was listed, the best hyperparameter value was selected using nested cross-validation (CV), using a grid search approach with a stratified 5-fold CV. The stacked generalization model consisted of a Linear SVM with heuristic C^61^ (model LinearSVMHC) for each type of neuroimaging feature (GMV, surface, fALFF, GCOR and LCOR) as the first level. The output of each of these five models was used as features of a second-level logistic regression model. For training the second-level model, the out-of-sample predictions of the first-level models were obtained using a stratified 5-fold CV scheme. An overview of the general methodological approach from brain images and questionnaires data to the evaluation of ML models is depicted in Fig. 1.

**Table 2:** List of models tested, including learning algorithms and hyperparameters evaluated

#	Name	Learning algorithm	Hyperparameter	Values
**1**	GSET	Extra trees	estimators	200, 500
			Criterion	Gini, entropy, log loss
			Max features	Sqrt, log2
**2**	GSRF	Random Forest	Estimators	200, 500
			Criterion	Gini, entropy, log loss
			Max features	Sqrt, log2
**3**	GSSVM-RBF	SVM	Kernel	Rbf
			C	1e^−4^, 1e^−3^, 1e^−2^, 1e^−1^, 1, 10, 100, 1e^4^, 1e^5^, 1e^6^
			Gamma	1e^−7^, 1e^−6^, 1e^−5^, 1e^−4^, 1e^−3^, 1e^−2^, 1e^−1^, 1, 10, 100, 1e^4^
**4**	GSLinearSVM	Linear SVM	C	1e^−4^, 1e^−3^, 1e^−2^, 1e^−1^, 1, 10, 100, 1e^4^, 1e^5^, 1e^6^
**5**	LinearSVMHC	Linear SVM	C	Heuristic^61^
			Dual	False
			Penalty	L1
**6**	LogitHC	Logit	C	Heuristic^61^
			Dual	False
			Penalty	L1

### Model evaluation

The available data was first split into 70% training and 30% hold-out test sets to avoid data leakage. Then, the generalization performance of the models (i.e. the capacity to generalize to unseen data) was evaluated on the training set using a stratified 5-fold cross-validation scheme, repeated five times, resulting in 25 evaluation runs. Finally, to validate the CV performance estimation, the models were retrained on the full training set and tested on the hold-out test set. To evaluate different aspects of model performance, such as the trade-off between specificity and sensitivity, we computed two threshold-dependent metrics, namely balanced accuracy and F1 score and two threshold-independent metrics, area under the receiver-operator characteristic (ROC) curve and average precision. Balanced accuracy is computed as the relative number of correct predictions over the total samples, weighted by the number of elements in each class, so that the chance level is set at 0.5 and 1 would mean a perfect classification. The F1 score is the harmonic mean between precision and recall.^62^ In short, it measures the model’s balanced ability to detect positives (recall = sensitivity) and to have high precision (= positive predictive value), that is, a low rate of false-positive detections. The area under the ROC curve (ROC-AUC) provides an aggregate measure of performance across all possible classification thresholds by plotting the true-positive rate (sensitivity) over the false-positive rate (1—specificity) for each threshold level. Shortly, ROC-AUC can be interpreted as the probability that, given two predictions, the model ranks them in the correct order. A perfect model with sensitivity and specificity being equal to 1 at all threshold levels will have a ROC-AUC of 1, while random guessing will result in ROC-AUC of 0.5.^62^ Given that ROC-AUC is skewed for imbalanced datasets, which is the case for all the SH dimensions (see Table 1), a more suitable metric is the area under the Precision-Recall curve,^63^ also known as average precision. This metric considers both recall and precision like the F1-score but across all thresholds as the ROC-AUC does. A perfect model will yield an average precision of 1, while chance levels depend on class balance.

To obtain reference values for each metric, we used the performance of two baseline models, which do not use the features but rely solely on the distribution of classes during training time. A first baseline model named *majority* always predicts the value of the most frequent class in the training set. A second baseline model named *chance* draws random predictions weighted by the number of training samples in each class. All models for each SH dimension were evaluated using the same 5 × 5 CV folds. We then used the corrected paired Student’s *t*-test for comparing the CV performance of the ML models^64^ and corrected for multiple comparisons (across models) using the Bonferroni method. All the analysis described was implemented using Julearn^65^ and Scikit-learn.^66^ The codes are available on GitHub: https://github.com/juaml/ukb_sleep_prediction.

### Testing for confounding bias

Given that the goal of the study is to evaluate the relationship between SH and brain structure and function, and knowing that some demographic variables are deeply encoded in brain-imaging data (i.e age^67^ and sex^68^), we also evaluated if the obtained results were strictly related to brain structure and function or other confounding factors might be leading the prediction. In a first approach, we employed the partial and full confounder statistical tests.^69^ This test, developed following the conditional independence testing framework,^70^ uses permutation testing to evaluate the independence between pairs of variables, given a potentially high-dimensional random variable that may contain confounding factors. The partial confounder test is used to evaluate if there is a partial confounder bias in the predictions. The null hypothesis states that there is no confounder bias in the data, given the target variable (i.e. predictions are independent from the confounder). If the *P*-value of the partial test is below the threshold (*P* < 0.05), then the null hypothesis can be rejected, indicating that there is an association between the predictions and the confounder. On the other hand, the full confounder test evaluates the null hypothesis that the model is entirely driven by the confounder. A *P*-value below the threshold (*P* < 0.05) indicates the model is not fully driven by the confounder. Both tests were parametrized with 1000 permutations and 50 steps for the Markov-chain Monte Carlo sampling.

In a second approach, we evaluated and compared the predictive performance of the same learning algorithms, but trained solely on two sets of confounds: age and sex, and the non-imaging derived phenotypes as defined by Alfaro-Almagro *et al*.^71^ to be able to identify the influence of such factors on predicting SH.

### Statistical analysis of demograpic differences

To assess whether demographic variables differed between groups for each target phenotype, we conducted post hoc group comparisons on age and sex distribution. Age differences were evaluated using Welch’s *t*-test, which does not assume equal variances between groups. Sex distribution was compared using a chi-squared (χ²) test of independence, and effect sizes were reported using Cohen’s *d* for age and φ coefficient for sex ratio differences. For interpretability, effect sizes exceeding |*d*| ≥ 0.20 or |φ| ≥ 0.10 were considered potentially meaningful.

## Results

**Figure 2:**
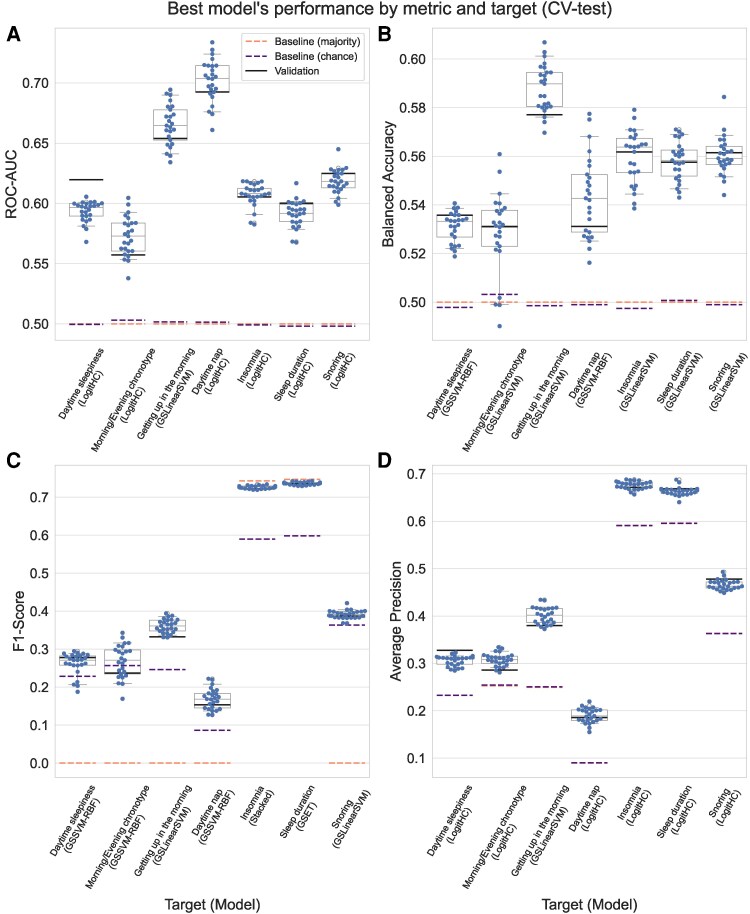
**Performances of the best model for each SH-related characteristic.** Each panel depicts the performance using a different metric: (**A**) Area under the receiver-operator characteristic (ROC-AUC), (**B**) balanced accuracy, (**C**) F1-Score and (**D**) average precision. Each blue dot represents the performance obtained at each of the 25 test folds within cross-validation (CV). Boxplots summarize the medians and 95% confidence intervals for the underlying distribution. As a reference, dashed orange lines depict the mean performance of a model that constantly predicts the most frequent class, purple lines depict the mean performance of a model that draws random predictions weighted by the number of samples in each class and black lines indicate the performance on the hold-out (validation) data.

We first trained and evaluated all seven models for each of the seven SH-related characteristics, a procedure that took 12.24 core-years, which is approximately 1.5 years on an eight-core desktop computer. For each SH-related characteristic and metric, we selected the best model among the seven competing models according to the performance of the respective metric upon evaluation on 5 × 5 = 25 CV-folds. This resulted in one model per SH-related characteristic and metric, which were then applied to the 30% hold-out test set. The performance of the best model for each SH-related characteristic and metric can be seen in Fig. 2. A complete description of the estimated performances for each metric can be seen in Supplementary Table 3.

When only considering the CV performance (which is commonly reported in research settings), some of the SH-related characteristics showed a modest predictability on several metrics. For instance, the best models for *insomnia* and *sleep duration* showed modest balanced accuracy (0.588 and 0.584) and AUC-ROC (0.549 and 0.553) and relatively high F1-score (0.725 and 0.739) and average precision (0.664 and 0.658). However, since some SH-related characteristics have imbalanced classes, it is important to note the performance of the baseline models. For example, the F1 score for insomnia and sleep duration is below the performance of the *majority baseline* model, meaning that a model that simply assigns the majority class to each sample showed a better F1 score. This is, indeed, due to the nature of the F1 scoring, in which chance-level depends on the ratio between classes. The limitation of AUC-ROC with imbalanced data also becomes clear for the *easiness getting up* characteristic, which showed a relatively high AUC-ROC but relatively lower average precision. Furthermore, as cross-validated performances could be overestimated,^72^ we evaluated the models on the hold-out data (30% of the samples). The obtained results fall within the confidence intervals of the CV-estimated performances (black lines in Fig. 1), suggesting that no over-estimation happened in our case. For more details on the values obtained for each model and SH-related characteristic, see Supplementary Table 4. Overall, our results indicate a weak predictive power but systematically above baseline models for each of the seven SH-related characteristics.

A common ML pitfall with a lack of predictive power is *overfitting*. This occurs when the model closely learns the idiosyncrasies of the training data, thus being incapable of making correct predictions on new, unseen samples. To verify that this is not the case, we computed the same metrics for each model but on the training samples. That is, how well each model memorized the training data. The results indicate that while some models were indeed overfitted, at least one model per SH-related characteristic was not (Supplementary Table 5). Given the comparable out-of-sample performance across models for each SH-related characteristic, and that the hyperparameters were selected in nested CV to prevent overfitting, we can safely conclude that overfitting is not a major issue in our results.

We then aimed to identify if the obtained results were purely brain-based predictions or the consequence of confounding bias. In other terms, evaluate if it is possible that the results are simply driven by variables such as the age and sex of the participants, which are known to affect brain structure and function. We employed two different statistical tests: the partial and full confounder tests.^69^ The partial confounder test results indicated that among the best models, all of them were partially driven by age and sex (*P* < 1e−3), except for the models predicting the Morning/Evening chronotype, which indicated that there is no evidence to claim that models are partially driven by confounds (*P* > 0.05). On the other hand, the full confounder test indicated that none of the models are fully driven by age and sex (*P* ≤ 0.001). The full list of *P*-values for each of the evaluated models can be seen in Supplementary Table 6.

**Figure 3:**
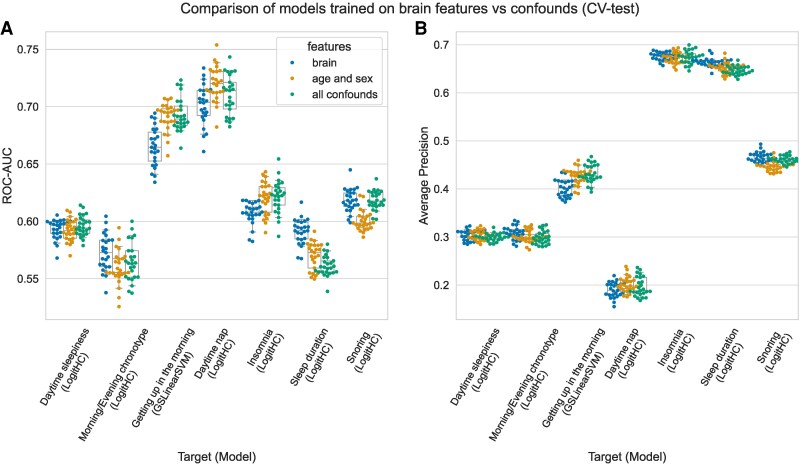
**Comparison of models trained on brain features versus confounds.** Side-by-side comparison of the best model for each SH-related characteristic using either brain features (blue), age and sex (orange) and all non-imaging-derived confounds (green) indicates that simple demographic variables have the same predictive capacity as complex neuroimaging data in the case of SH-related characteristics. Each panel represents a threshold-free metric: (**A**) area under the receiver-operator characteristic curve and (**B**) average precision. Each dot represents the performance obtained at each of the 25 test folds within cross-validation (CV). Boxplots summarize the medians and 95% confidence intervals for the underlying distribution.

To assess the extent to which age and sex influence the predictions, we trained the learning algorithms from the best models using only these two variables as features. The results, obtained after 13.14 core-years, are depicted in Fig. 3. In short, the age and sex models, as well as the full set of non-imaging-derived confounds, perform similarly for most of the SH-related characteristics, with the exceptions of sleep duration, whose prediction by confounds was worse than the prediction by brain features, and ‘Easiness Getting up in the Morning’ and ‘Daytime Nap’ for which the inverse was true. It is important to note that these models were not optimized for age and sex or the full confounds set but uses the same hyper parametrization as for brain features to serve as a comparison. The full panel, including the threshold-dependent metrics, is depicted in Supplementary Fig. 1.

To contextualize model performance, we conducted a post hoc demographic analysis to assess whether age and sex distributions differed across target groups. The results revealed that several target phenotypes exhibited statistically significant group differences in age (Supplementary Table 7) and/or sex distribution (Supplementary Table 8), suggesting that these demographic variables carry a meaningful predictive signal that may partially explain ML model performance. For sleep duration, the age difference between groups was small but statistically significant (*t* = 13.13, *P* = 3.84 × 10^−39^, *d* = 0.21). More pronounced age effects were observed for getting up in the morning (*t* = −28.98, *P* = 2.02 × 10^−172^, *d* = 0.58), Daytime nap (*t* = 21.12, *P* = 5.05 × 10⁻⁸⁹, d = 0.56) and daytime sleepiness (*t* = 20.94, *P* = 1.94 × 10⁻⁹⁵, *d* = 0.30), indicating moderate-to-large age-related shifts between positive and negative subgroups. Morning/evening chronotype also showed a statistically reliable age effect, though with a borderline effect size (*t* = −8.36, *P* = 8.24 × 10^−17^, *d* = −0.20).

With respect to sex distribution, large deviations in sex ratio were observed for insomnia (χ² = 581.14, *P* = 2.12 × 10^−128^, φ = 0.20), getting up in the morning (χ² = 471.33, *P* = 1.64 × 10^−104^, φ = 0.18), daytime nap (χ² = 475.70, *P* = 1.84 × 10^−105^, φ = 0.16) and snoring (χ² = 699.36, *P* = 4.12 × 10^−154^, φ = 0.16), indicating systematic sex-related bias in target group composition. Together, these results demonstrate that age and sex carry non-trivial discriminative value for several phenotypes, supporting the interpretation that ML models trained on demographic features alone may achieve above-chance performance due to these underlying population-level structure differences.

## Discussion

The current large-scale study systematically evaluated ML-based predictive analysis for classifying extremes of seven different SH-related characteristics based on multiple neuroimaging markers in UKB. We covered a large range of local and global multi-modal neuroimaging features covering brain structure and function and employed several ML algorithms in a nested cross-validation setting and a hold-out test set evaluated on four metrics (balanced accuracy, average precision, ROC-AUC, F1-score). Our striking findings demonstrated that the balanced accuracy for predicting SH-related characteristics did not exceed 56%, which indicates that brain structure and function measures could not accurately predict any of the SH-related characteristics. The slight improvement over baseline models across the evaluation metrics suggests that the ML algorithms indeed captured some underlying patterns in the data. However, we do not consider these results as high predictive accuracy compared to other behavioral or neuroimaging-based predictions, such as sex,^68,73^ neurodegenerative diseases^74^ and depressive symptoms severity.^42^ Furthermore, the comparable predictive performance observed in models trained only on age and sex might be why brain-based models can predict above-chance levels. Put differently, we did not observe strong evidence to claim that the brain measures can predict SH-related characteristics independently of age and sex or a previously selected set of non-imaging derived variables.^71^ In the following, we discuss the potential reasons for the poor efficacy of multi-modal brain features in predicting SH-related characteristics in UKB.

### Target issues: SH is a heterogenous concept

Our findings align with previous large-scale sample studies using e.g. UKB and ENIGMA-Sleep datasets that did not observe a robust association between brain structure and insomnia symptoms^8,23^ and sleep duration.^4^ SH has a heterogeneous definition across different general population datasets, as well as clinical samples. Although some studies used a standard sleep questionnaire such as the Pittsburgh Sleep Quality Index (PSQI) to assess sleep quality or the Regulatory Satisfaction Alertness Timing Efficiency Duration (RU-SATED) questionnaire as a valid measure of SH,^75^ the UKB did not use those standard questionnaires. Instead, seven self-reported questions were provided about sleep duration, difficulties in getting up in the morning, chronotype, nap, daytime sleepiness and two measures of clinical conditions such as insomnia symptoms and snoring. Considering these single questions for various SH domains could have affected the clarity and meaningfulness of the measured SH characteristics. Furthermore, the accuracy of self-report sleep assessment based on single items and selective participation or recall biases to answer those questions could have led to measurement issues, which have been highlighted previously.^76^

Another critical aspect is differentiating the sleep-related symptoms of insomnia and snoring in the general population from clinical conditions. It is well-documented that insomnia disorder is a heterogeneous condition with different subtypes with noticeable inconsistencies in terms of pathophysiology, symptomatology and treatment response.^8,16,18,26,77-80^ According to the third edition of the International Classification of Sleep Disorders (ICSD-3),^81^ significant daytime dysfunction and having adequate opportunity and circumstances to sleep are essential diagnostic criteria for insomnia disorder. Thus, relying on a single question, i.e. ‘Do you have trouble falling asleep at night or do you wake up in the middle of the night?’ Cannot capture this 24-hour phenomenon and can be misunderstood by nocturia, which is common in older adults. Similarly, snoring can have several etiologies beyond it being a cardinal symptom of OSA, including genetic factors, obesity, nasal blockages, alcohol abuse, smoking or medications.^82^ Thus, these limited questions are not sufficient to define clinical insomnia disorder or OSA.

Additionally, the imbalance in target labels influences model performance, hindering the learning of sufficient information for accurate classification. Particularly for SH-related characteristics such as ‘Easiness Getting up in the Morning’, ‘Day Naps’ and ‘Daytime Dozing,’ the uneven distribution of target labels has resulted in models achieving moderate ROC-AUC scores around 0.6, while the balanced accuracy remained at the chance level of approximately 0.5. This discrepancy between ROC-AUC and balanced accuracy highlights the challenges in achieving fairness and robustness in the models’ predictive capabilities when dealing with imbalanced target datasets. An imbalanced target affects both the learning and interpretation of threshold-dependent metrics.^83^ Thus, our conclusions regarding the limited predictive capacity are based on the ROC-AUC and average precision metrics, which are threshold-independent and have been suggested to be preferable for drawing scientific conclusions.^63,84^

The SH-related characteristics in the UKB sample do not represent cross-country sleep differences well. Data from 63 countries showed that individuals from East Asia tend to sleep less and participants from East Europe report longer sleep duration.^85^ Similarly, another study on ∼220 000 wearable device users in 35 countries observed shorter sleep duration, later sleep timing and less sleep efficiency in East Asia compared with Western Europe, North America and Oceania, probably due to social- and work-related cultural differences regarding the coping with inadequate sleep and sleep debt.^86^ Moreover, there are significant differences in daytime napping across cultures, being more common in non-Western countries.^86^ Notably, approximately 10% of the UKB participants reported regular daily naps (Table 1).

### Input features issues: regional brain measurements

Our results also suggest that the neuroimaging features applied in our study may not capture the full spectrum of brain-related features relevant to SH or that the selected features may not be sensitive enough to the subtleties of SH. Moreover, it raises the possibility that current feature sets are insufficiently granular to mirror the complex biological underpinnings of SH. The low performance of the models in predicting SH dimensions, therefore, points to the need for a deeper investigation into more sensitive and comprehensive neuroimaging metrics that can better encapsulate the factors influencing SH. SH might be associated with brain circuits that can be captured, e.g. via seed-based structural or FC measures rather than local brain abnormalities that we used from brain parcels, including GMV, grey/white matter contrast, pial surface, white matter surface, white matter thickness and white matter volume, LCOR and fALFF. It has been reported that insomnia symptoms were associated with higher FC within the DMN and FPN and lower FC between the DMN and SN.^18^ Wang and colleagues also found that SH dimensions are correlated with disrupted FC patterns in the attentional and thalamic networks in several datasets.^7^ Another study using UKB data found associations between SH and FC and structural connectivity. Within-network hyperconnectivity in DMN, FPN and SN has been observed in healthy subjects and patients with mild cognitive impairment with insomnia symptoms, while patients with Alzheimer’s disease and insomnia symptoms showed hypoconnectivity in those networks.^17^ Although we included GCOR, representing functional correlations between a given voxel and other brain voxels (i.e. degree centrality), it did not improve the prediction when used as an input with local markers together. Recently, Lynch *et al*. performed 62 repeated neuroimaging measurements in major depressive disorder (MDD). Using precision functional mapping, they identified that long-term FC changes in the frontostriatal circuits can predict future depressive symptoms.^87^ Thus, future studies could explore network-based and white matter integrity metrics as input features or longitudinal precision functional mapping to predict SH in UKB.

Our results also remind us to think beyond the brain feature modalities. Recently, we observed that sleep quality and anxiety robustly predict depressive symptom severity across three independent datasets. Still, brain structural and functional features could not predict depressive symptoms, which indicated that parcellated brain imaging data may not be beneficial in predicting mental health.^42^ A large-scale study by the ENIGMA-Anxiety Consortium utilized ML to analyse neuroanatomical data for youth anxiety disorders and also achieved only modest classification accuracy (AUC 0.59–0.63).^88^ This parallels findings from extensive ML optimization efforts with MDD, which observed mean accuracies in distinguishing patients from controls that ranged from 48.1% to 62.0% only, even when additionally provided with polygenic risk scores, casting doubt on the potential diagnostic relevance of neuroimaging and genetic biomarkers for MDD.^89^ Similarly, the ENIGMA-MDD consortium's multi-site study^90^ achieved a balanced accuracy of only about 62% in classifying MDD versus healthy controls, which further dropped to approximately 52% after harmonization for site effect. Random chance accuracy was also observed across various stratified groups. These findings may point to an alternative view that complex psychiatric conditions such as sleep disturbance or depression represent deficits in the brain–body interaction, which suggests that body organ health measurements, such as metabolic and cardiovascular systems, in addition to brain imaging, should be considered.^91,92^

### ML-related issues

Following proper ML pipelining practices such as nested CV and grid search for meticulous hyperparameter tuning—methods that typically enhance a model's capacity to generalize—our models did not achieve high predictive performance. Our study's low classification performance highlights the inherent challenges in developing models that accurately capture the complex nature of SH using brain imaging data. ML models are designed to discern patterns and generalize findings to new, unseen data. However, like any statistical analysis, ML is challenged when the target labels are unreliable.^93^ We reduced the uncertainty in the labeling to some degree by using extreme values for each SH-related characteristic. This should make learning easier for the ML algorithms and boost accuracy. The low performance observed despite this simplification suggests that the prediction of SH-related characteristics as a continuum could be more challenging. Difficulty in creating generalizable ML models arises from potential heterogeneity in how SH is reflected in the brain. In this case, the ML models will not be able to learn a consistent pattern, leading to low performance. Further analysis of SH subtypes and more refined scales are needed to discern this possibility. Finally, several of our classification tasks were imbalanced, i.e. one of the classes was much more frequently present than the other. Such an imbalance can lead to biased ML models, which in turn lack generalization ability. To this end, we employed AUC-ROC and average precision metrics to evaluate the ML pipelines. These metrics are independent of a threshold used for dichotomization and thus suitable for characterizing the performance in imbalanced datasets, particularly with tree ensemble models.^83^

### Strengths, limitations and future directions

The present study has several advantages over other case–control SH-brain studies. Here, we calculated 4677 structural and functional brain features as input features from 28 088 participants from the UKB and applied several ML algorithms to classify the extremes of seven SH-related characteristics. In particular, (i) including diverse and multi-modal neuroimaging metrics is crucial. Multi-modal data enriches the ML analysis, allowing for a more comprehensive exploration and interpretation of the neurobiological correlates of SH at both structural and functional levels; (ii) we leveraged the detailed features provided by the Schaefer atlas (1000 ROIs), which is supported by our ample sample size. This approach assumes that if relevant information is present in an ROI, our models—given their complexity—are equipped to detect it, whether the information is concentrated within a single ROI or dispersed across several regions; (iii) we carefully designed our ML analyses using fully separated train and test samples to avoid any leakage of the test set into the model, which is a common oversight in some ML studies^94^; (iv) the ML analyses were conducted using several rather different algorithms including Random Forest, Extremely Randomized Trees, support vector machine, logistic regression and stacked generalization; (v) we applied a grid search-based hyperparameter optimization to prevent overfitting and increase the generalizability of our findings.

Our results should be interpreted within the context of the study's limitations and the nascent state of this field. This study did not include any objective sleep assessment such as polysomnography. Although polysomnography is recommended as a gold-standard objective measure for diagnosing several sleep disorders, including OSA, its validity for insomnia or sleep quality assessment remains disputed.^95^ Moreover, some evidence showed only a weak association between the subjective sleep measurement (e.g. PSQI) and polysomnography in patients with insomnia disorder.^96^ Here, we focussed on self-reported information on SH. Thus, future studies should consider performing an ML analysis of objective sleep data and comparing it with the analysis of subjective data^97^ yet subjective data is usually confounded with other lifestyle factors, which are not necessarily linked to brain structure and function.^98^ Future studies could apply normative modelling, a technique that studies deviations from population norms to show the range of inter-individual differences in brain structure. Unlike traditional case–control paradigms that rely on common neurobiological factors across all subjects, normative modelling focuses on individual deviations from normal patterns, making it a promising approach to consider inter-individual variability in brain expression of SH.^92,99,100^ Furthermore, one can also employ longitudinal and objective sleep measures of UKB, such as accelerometry and follow-up imaging data, to add valuable depth to our analyses. Longitudinal studies can help identify the long-term interaction between the SH and the brain together with well-characterized sleep measurements from collaborative research groups, e.g. the ENIGMA-Sleep consortium,^101^ to provide replicable results across different countries.

## Conclusion

The present extensive ML study using a large population sample demonstrated that multi-modal neuroimaging markers had low efficacy in separating the extremes of various SH-related characteristics in the UKB. This suggests that the interaction between SH and brain organization may be more complex to be captured with the current ML models and neuroimaging features. While our methodological approach is comprehensive and aims to establish links between neuroimaging features and SH dimensions, this study acknowledges the complexity of interpreting neuroimaging in the context of SH. We need future cross-sectional and longitudinal studies considering brain circuits, objective sleep measurements and cross-country sleep assessments to evaluate the sophisticated brain-sleep interplay.

## Supplementary Material

fcag016_Supplementary_Data

## Data Availability

This research has been conducted using data from the UK Biobank resources (application number 41655). All data used in this study are publicly accessible from the UK Biobank via their standard data access procedure (http://www.ukbiobank.ac.uk/). Due to the UK Biobank policy, no derivatives of such can be shared as they contain identifiable information. All codes used in the development of this research can be found online at https://github.com/juaml/ukb_sleep_prediction.
