# Supplementary material for: Can we predict sleep health based on brain features? A large-scale machine learning study using the UK Biobank
Source: Brain Commun. 2026 Jan 22;8(1):fcag016. doi: 10.1093/braincomms/fcag016 (PMC12887735; doi:10.1093/braincomms/fcag016)
Supplement: fcag016_Supplementary_Data [file fcag016_supplementary_data.docx]

# Supplementary Information

**Can we predict sleep health based on brain features? A large-scale machine learning study using UK Biobank**

| **Sample Demographics** |  |  | N=28088 |
| --- | --- | --- | --- |
| Sex | Female | 14860 | 52.90% |
|  | Male | 13228 | 47.09% |
|  | Ratio (M/F) | 0.89 |  |
| Age (years) | Mean | 64.1 |  |
|  | Std | 7.54 |  |
|  | Min | 44.58 |  |
|  | Max | 82.25 |  |
| Years of Education | Mean | 16.71 |  |
|  | Std | 3.75 |  |
|  | Min | 7 |  |
|  | Max | 19 |  |
| Ethnic Background | Asian or Asian British | 9543 | 1.08% |
|  | Black or Black British | 2398 | 0.65% |
|  | Chinese | 87 | 0.30% |
|  | Mixed | 125 | 0.44% |
|  | White | 27162 | 96.70% |
|  | Other Ethnic group | 149 | 0.53% |
|  | Do not know | 3 | 0.01% |
|  | Prefer not to answer | 63 | 0.22% |
| Self-reported health rating | Excellent | 6747 | 24.02% |
|  | Good | 16924 | 60.25% |
|  | Fair | 3868 | 13.77% |
|  | Poor | 497 | 1.76% |
|  | Do not know | 41 | 0.14% |
|  | Prefer not to answer | 1 | 0.003% |

Supplementary Table 1. Demographic variables including sex, age (in years), years of education, ethnic background and self-reported health rating from the 28,088 samples from which the MRI-related features were extracted.

| Features | Atlas | # ROIs | # Samples |
| --- | --- | --- | --- |
| GMV | Schaefer | 1000 | 39316 |
|  | Melbourne Subcortical | 54 | 39172 |
|  | Diedrichsen | 34 | 38490 |
| gray/white matter contrast | Desikan-Kiliany | 68 | 43107 |
| Pial surface | Desikan-Kiliany | 64 | 43107 |
| White matter surface | Desikan-Kiliany | 66 | 43107 |
| White matter thickness | Desikan-Kiliany | 66 | 43107 |
| White matter volume | Desikan-Kiliany | 64 | 43107 |
| fALFF | Schaefer | 1000 | 29367 |
|  | Melbourne Subcortical | 54 | 29367 |
|  | Diedrichsen | 33 | 29367 |
| LCOR | Schaefer | 1000 | 29367 |
|  | Melbourne Subcortical | 54 | 29367 |
|  | Diedrichsen | 33 | 29367 |
| GCOR | Schaefer | 1000 | 29367 |
|  | Melbourne Subcortical | 54 | 29367 |
|  | Diedrichsen | 33 | 29367 |

Supplementary Table 2. List of features extracted from functional and structural brain imaging, indicating the parcellation applied, the number of ROIs extracted, and the number of participants for which the features were successfully extracted. Note: GMV = Gray Matter Volume, fALFF = Fractional Amplitude of Low-Frequency Fluctuations, LCOR = Local Correlation, GCOR = Global Correlation.

| SH Characteristic | Model | ROC AUC | Balanced Accuracy | F1-Score | Average Precision |
| --- | --- | --- | --- | --- | --- |
| Daytime Sleepiness | Extra Trees | 0.581 ± 0.009 | 0.5 ± 0.0 | 0.001 ± 0.001 | 0.288 ± 0.008 |
|  | Linear SVM | 0.562 ± 0.014 | 0.527 ± 0.007 | 0.23 ± 0.056 | 0.278 ± 0.011 |
|  | Random Forest | 0.575 ± 0.007 | 0.501 ± 0.001 | 0.005 ± 0.004 | 0.282 ± 0.006 |
|  | SVM (RBF Kernel) | 0.558 ± 0.009 | **0.531 ± 0.006** | **0.262 ± 0.03** | 0.275 ± 0.005 |
|  | Linear SVM (Heuristic C) | 0.581 ± 0.007 | 0.514 ± 0.003 | 0.1 ± 0.01 | 0.295 ± 0.01 |
|  | Logit (Heuristic C) | **0.593 ± 0.009** | 0.501 ± 0.001 | 0.008 ± 0.003 | **0.305 ± 0.011** |
|  | Stacked | 0.511 ± 0.004 | 0.5 ± 0.0 | 0.002 ± 0.003 | 0.24 ± 0.004 |
| Morning/  Evening chronotype | Extra Trees | 0.539 ± 0.021 | 0.5 ± 0.002 | 0.004 ± 0.005 | 0.287 ± 0.016 |
|  | Linear SVM | 0.557 ± 0.025 | **0.529 ± 0.017** | 0.262 ± 0.022 | 0.299 ± 0.022 |
|  | Random Forest | 0.538 ± 0.019 | 0.501 ± 0.002 | 0.01 ± 0.009 | 0.282 ± 0.014 |
|  | SVM (RBF Kernel) | 0.54 ± 0.018 | 0.521 ± 0.016 | **0.268 ± 0.045** | 0.284 ± 0.016 |
|  | Linear SVM (Heuristic C) | 0.549 ± 0.019 | 0.511 ± 0.007 | 0.112 ± 0.023 | 0.292 ± 0.015 |
|  | Logit (Heuristic C) | **0.572 ± 0.017** | 0.5 ± 0.0 | 0.0 ± 0.0 | **0.308 ± 0.014** |
|  | Stacked | 0.512 ± 0.009 | 0.5 ± 0.0 | 0.0 ± 0.001 | 0.26 ± 0.006 |
| Easiness getting up in the morning | Extra Trees | 0.631 ± 0.02 | 0.502 ± 0.002 | 0.011 ± 0.007 | 0.36 ± 0.02 |
|  | Linear SVM | **0.665 ± 0.018** | **0.588 ± 0.01** | **0.361 ± 0.018** | **0.401 ± 0.018** |
|  | Random Forest | 0.619 ± 0.017 | 0.504 ± 0.003 | 0.026 ± 0.009 | 0.344 ± 0.017 |
|  | SVM (RBF Kernel) | 0.634 ± 0.02 | 0.57 ± 0.006 | 0.324 ± 0.014 | 0.372 ± 0.017 |
|  | Linear SVM (Heuristic C) | 0.653 ± 0.017 | 0.553 ± 0.008 | 0.25 ± 0.019 | 0.383 ± 0.016 |
|  | Logit (Heuristic C) | 0.664 ± 0.015 | 0.515 ± 0.005 | 0.074 ± 0.018 | 0.396 ± 0.016 |
|  | Stacked | 0.563 ± 0.01 | 0.515 ± 0.005 | 0.076 ± 0.021 | 0.302 ± 0.011 |
| Daytime nap | Extra Trees | 0.631 ± 0.02 | 0.502 ± 0.002 | 0.011 ± 0.007 | 0.36 ± 0.02 |
|  | Linear SVM | **0.665 ± 0.018** | **0.588 ± 0.01** | **0.361 ± 0.018** | **0.401 ± 0.018** |
|  | Random Forest | 0.619 ± 0.017 | 0.504 ± 0.003 | 0.026 ± 0.009 | 0.344 ± 0.017 |
|  | SVM (RBF Kernel) | 0.634 ± 0.02 | 0.57 ± 0.006 | 0.324 ± 0.014 | 0.372 ± 0.017 |
|  | Linear SVM (Heuristic C) | 0.653 ± 0.017 | 0.553 ± 0.008 | 0.25 ± 0.019 | 0.383 ± 0.016 |
|  | Logit (Heuristic C) | 0.664 ± 0.015 | 0.515 ± 0.005 | 0.074 ± 0.018 | 0.396 ± 0.016 |
|  | Stacked | 0.563 ± 0.01 | 0.515 ± 0.005 | 0.076 ± 0.021 | 0.302 ± 0.011 |
| Insomnia | Extra Trees | 0.59 ± 0.012 | 0.531 ± 0.007 | 0.724 ± 0.005 | 0.661 ± 0.013 |
|  | Linear SVM | 0.588 ± 0.012 | **0.56 ± 0.011** | 0.661 ± 0.009 | 0.663 ± 0.01 |
|  | Random Forest | 0.59 ± 0.012 | 0.535 ± 0.007 | 0.721 ± 0.007 | 0.662 ± 0.011 |
|  | SVM (RBF Kernel) | 0.595 ± 0.016 | 0.551 ± 0.007 | 0.709 ± 0.016 | 0.668 ± 0.015 |
|  | Linear SVM (Heuristic C) | 0.578 ± 0.009 | 0.55 ± 0.008 | 0.671 ± 0.006 | 0.655 ± 0.009 |
|  | Logit (Heuristic C) | **0.607 ± 0.01** | 0.553 ± 0.008 | 0.717 ± 0.006 | **0.676 ± 0.009** |
|  | Stacked | 0.583 ± 0.009 | 0.535 ± 0.006 | **0.727 ± 0.004** | 0.647 ± 0.007 |
| Sleep duration | Extra Trees | 0.572 ± 0.014 | 0.531 ± 0.006 | **0.736 ± 0.004** | 0.645 ± 0.011 |
|  | Linear SVM | 0.587 ± 0.008 | **0.558 ± 0.008** | 0.672 ± 0.008 | 0.663 ± 0.009 |
|  | Random Forest | 0.573 ± 0.014 | 0.532 ± 0.007 | 0.734 ± 0.005 | 0.648 ± 0.012 |
|  | SVM (RBF Kernel) | 0.575 ± 0.014 | 0.544 ± 0.006 | 0.682 ± 0.035 | 0.656 ± 0.011 |
|  | Linear SVM (Heuristic C) | 0.581 ± 0.008 | 0.551 ± 0.008 | 0.681 ± 0.009 | 0.657 ± 0.01 |
|  | Logit (Heuristic C) | **0.591 ± 0.011** | 0.545 ± 0.005 | 0.731 ± 0.006 | **0.664 ± 0.008** |
|  | Stacked | 0.57 ± 0.009 | 0.526 ± 0.004 | 0.735 ± 0.005 | 0.641 ± 0.008 |
| Snoring | Extra Trees | 0.574 ± 0.012 | 0.503 ± 0.002 | 0.032 ± 0.006 | 0.423 ± 0.011 |
|  | Linear SVM | 0.6 ± 0.009 | **0.56 ± 0.007** | **0.391 ± 0.011** | 0.45 ± 0.01 |
|  | Random Forest | 0.571 ± 0.012 | 0.505 ± 0.003 | 0.054 ± 0.012 | 0.42 ± 0.013 |
|  | SVM (RBF Kernel) | 0.576 ± 0.008 | 0.547 ± 0.007 | 0.378 ± 0.012 | 0.428 ± 0.009 |
|  | Linear SVM (Heuristic C) | 0.592 ± 0.008 | 0.551 ± 0.006 | 0.356 ± 0.011 | 0.444 ± 0.011 |
|  | Logit (Heuristic C) | **0.618 ± 0.01** | 0.538 ± 0.005 | 0.234 ± 0.01 | **0.466 ± 0.011** |
|  | Stacked | 0.578 ± 0.008 | 0.52 ± 0.005 | 0.138 ± 0.018 | 0.425 ± 0.009 |

Supplementary Table 3: Results of the out-of-sample evaluation (CV) of the 7 models for each SH-related characteristic. Values represent the mean and standard deviation across CV repetitions. Values in bold represent the best performance for each metric and SH-related characteristic across the 7 models.

| SH Characteristic | Model | ROC AUC | Balanced Accuracy | F1-Score | Average Precision |
| --- | --- | --- | --- | --- | --- |
| Daytime Sleepiness | Random Forest | 0.501 | 0.006 | 0.289 | 0.585 |
|  | Linear SVM (Heuristic C) | 0.512 | 0.087 | 0.297 | 0.589 |
|  | Linear SVM | 0.53 | 0.262 | 0.266 | 0.547 |
|  | Logit (Heuristic C) | 0.502 | 0.012 | 0.327 | 0.62 |
|  | SVM (RBF Kernel) | 0.536 | 0.278 | 0.274 | 0.559 |
|  | Stacked | 0.501 | 0.006 | 0.238 | 0.51 |
|  | Extra Trees | 0.499 | 0.0 | 0.258 | 0.524 |
| Morning/  Evening chronotype | Random Forest | 0.5 | 0.006 | 0.256 | 0.527 |
|  | Linear SVM (Heuristic C) | 0.505 | 0.09 | 0.289 | 0.564 |
|  | Linear SVM | 0.531 | 0.232 | 0.291 | 0.571 |
|  | Logit (Heuristic C) | 0.5 | 0.0 | 0.286 | 0.557 |
|  | SVM (RBF Kernel) | 0.53 | 0.237 | 0.286 | 0.555 |
|  | Stacked | 0.5 | 0.0 | 0.253 | 0.515 |
|  | Extra Trees | 0.5 | 0.006 | 0.342 | 0.622 |
| Easiness getting up in the morning | Random Forest | 0.502 | 0.025 | 0.331 | 0.61 |
|  | Linear SVM (Heuristic C) | 0.549 | 0.24 | 0.362 | 0.639 |
|  | Linear SVM | 0.577 | 0.333 | 0.38 | 0.654 |
|  | Logit (Heuristic C) | 0.518 | 0.089 | 0.39 | 0.663 |
|  | SVM (RBF Kernel) | 0.555 | 0.3 | 0.338 | 0.609 |
|  | Stacked | 0.515 | 0.08 | 0.296 | 0.561 |
|  | Extra Trees | 0.5 | 0.0 | 0.169 | 0.666 |
| Daytime nap | Random Forest | 0.501 | 0.004 | 0.154 | 0.643 |
|  | Linear SVM (Heuristic C) | 0.5 | 0.0 | 0.163 | 0.668 |
|  | Linear SVM | 0.525 | 0.146 | 0.111 | 0.583 |
|  | Logit (Heuristic C) | 0.501 | 0.004 | 0.186 | 0.693 |
|  | SVM (RBF Kernel) | 0.531 | 0.154 | 0.113 | 0.586 |
|  | Stacked | 0.5 | 0.0 | 0.09 | 0.5 |
|  | Extra Trees | 0.535 | 0.719 | 0.661 | 0.586 |
| Insomnia | Random Forest | 0.542 | 0.717 | 0.653 | 0.587 |
|  | Linear SVM (Heuristic C) | 0.545 | 0.659 | 0.655 | 0.58 |
|  | Linear SVM | 0.562 | 0.657 | 0.662 | 0.587 |
|  | Logit (Heuristic C) | 0.551 | 0.703 | 0.672 | 0.605 |
|  | SVM (RBF Kernel) | 0.555 | 0.705 | 0.667 | 0.599 |
|  | Stacked | 0.536 | 0.722 | 0.657 | 0.597 |
|  | Extra Trees | 0.533 | 0.735 | 0.646 | 0.579 |
| Sleep duration | Random Forest | 0.532 | 0.735 | 0.652 | 0.581 |
|  | Linear SVM (Heuristic C) | 0.554 | 0.686 | 0.663 | 0.586 |
|  | Linear SVM | 0.558 | 0.679 | 0.672 | 0.596 |
|  | Logit (Heuristic C) | 0.542 | 0.728 | 0.669 | 0.6 |
|  | SVM (RBF Kernel) | 0.55 | 0.664 | 0.649 | 0.568 |
|  | Stacked | 0.539 | 0.742 | 0.651 | 0.589 |
|  | Extra Trees | 0.503 | 0.035 | 0.428 | 0.579 |
| Snoring | Random Forest | 0.507 | 0.056 | 0.427 | 0.574 |
|  | Linear SVM (Heuristic C) | 0.552 | 0.355 | 0.453 | 0.606 |
|  | Linear SVM | 0.561 | 0.387 | 0.461 | 0.613 |
|  | Logit (Heuristic C) | 0.542 | 0.255 | 0.478 | 0.625 |
|  | SVM (RBF Kernel) | 0.551 | 0.375 | 0.444 | 0.595 |
|  | Stacked | 0.524 | 0.171 | 0.431 | 0.582 |
|  | Random Forest | 0.501 | 0.006 | 0.289 | 0.585 |

Supplementary Table 4: Results of the validation sample evaluation of the 7 models for each SH-related characteristic.

| SH Characteristic | Model | ROC AUC | Balanced Accuracy | F1-Score | Average Precision |
| --- | --- | --- | --- | --- | --- |
| Daytime Sleepiness | Extra Trees | 1.0 ± 0.0 | 1.0 ± 0.0 | 1.0 ± 0.0 | 1.0 ± 0.0 |
|  | Linear SVM | 0.871 ± 0.039 | 0.701 ± 0.068 | 0.548 ± 0.136 | 0.71 ± 0.074 |
|  | Random Forest | 1.0 ± 0.0 | 1.0 ± 0.0 | 1.0 ± 0.0 | 1.0 ± 0.0 |
|  | SVM (RBF Kernel) | 0.958 ± 0.058 | 0.919 ± 0.105 | 0.891 ± 0.145 | 0.923 ± 0.108 |
|  | Linear SVM (Heuristic C) | 0.799 ± 0.002 | 0.56 ± 0.003 | 0.222 ± 0.008 | 0.577 ± 0.004 |
|  | Logit (Heuristic C) | 0.668 ± 0.002 | 0.502 ± 0.001 | 0.011 ± 0.004 | 0.381 ± 0.004 |
|  | Stacked | 0.537 ± 0.003 | 0.502 ± 0.001 | 0.007 ± 0.005 | 0.274 ± 0.004 |
| Morning/  Evening chronotype | Extra Trees | 1.0 ± 0.0 | 1.0 ± 0.0 | 1.0 ± 0.0 | 1.0 ± 0.0 |
|  | Linear SVM | 0.906 ± 0.048 | 0.786 ± 0.092 | 0.698 ± 0.134 | 0.799 ± 0.101 |
|  | Random Forest | 1.0 ± 0.0 | 1.0 ± 0.0 | 1.0 ± 0.0 | 1.0 ± 0.0 |
|  | SVM (RBF Kernel) | 0.983 ± 0.023 | 0.931 ± 0.084 | 0.916 ± 0.104 | 0.974 ± 0.035 |
|  | Linear SVM (Heuristic C) | 0.858 ± 0.004 | 0.598 ± 0.008 | 0.333 ± 0.02 | 0.702 ± 0.009 |
|  | Logit (Heuristic C) | 0.637 ± 0.007 | 0.5 ± 0.0 | 0.0 ± 0.0 | 0.369 ± 0.007 |
|  | Stacked | 0.619 ± 0.024 | 0.5 ± 0.0 | 0.001 ± 0.002 | 0.404 ± 0.025 |
| Easiness getting up in the morning | Extra Trees | 1.0 ± 0.0 | 1.0 ± 0.0 | 1.0 ± 0.0 | 1.0 ± 0.0 |
|  | Linear SVM | 0.874 ± 0.003 | 0.735 ± 0.006 | 0.619 ± 0.009 | 0.724 ± 0.006 |
|  | Random Forest | 1.0 ± 0.0 | 1.0 ± 0.0 | 1.0 ± 0.0 | 1.0 ± 0.0 |
|  | SVM (RBF Kernel) | 0.989 ± 0.016 | 0.962 ± 0.048 | 0.953 ± 0.061 | 0.98 ± 0.027 |
|  | Linear SVM (Heuristic C) | 0.865 ± 0.004 | 0.651 ± 0.007 | 0.467 ± 0.015 | 0.707 ± 0.007 |
|  | Logit (Heuristic C) | 0.713 ± 0.005 | 0.522 ± 0.004 | 0.095 ± 0.014 | 0.458 ± 0.006 |
|  | Stacked | 0.661 ± 0.01 | 0.54 ± 0.011 | 0.155 ± 0.04 | 0.442 ± 0.012 |
| Daytime nap | Extra Trees | 1.0 ± 0.0 | 1.0 ± 0.0 | 1.0 ± 0.0 | 1.0 ± 0.0 |
|  | Linear SVM | 1.0 ± 0.0 | 0.999 ± 0.002 | 0.999 ± 0.002 | 1.0 ± 0.0 |
|  | Random Forest | 1.0 ± 0.0 | 1.0 ± 0.0 | 1.0 ± 0.0 | 1.0 ± 0.0 |
|  | SVM (RBF Kernel) | 1.0 ± 0.0 | 0.999 ± 0.001 | 0.999 ± 0.001 | 1.0 ± 0.001 |
|  | Linear SVM (Heuristic C) | 0.89 ± 0.003 | 0.503 ± 0.002 | 0.014 ± 0.006 | 0.546 ± 0.012 |
|  | Logit (Heuristic C) | 0.737 ± 0.005 | 0.5 ± 0.0 | 0.0 ± 0.0 | 0.223 ± 0.007 |
|  | Stacked | 0.501 ± 0.003 | 0.5 ± 0.0 | 0.0 ± 0.0 | 0.093 ± 0.004 |
| Insomnia | Extra Trees | 1.0 ± 0.0 | 1.0 ± 0.0 | 1.0 ± 0.0 | 1.0 ± 0.0 |
|  | Linear SVM | 0.82 ± 0.002 | 0.732 ± 0.003 | 0.796 ± 0.002 | 0.862 ± 0.002 |
|  | Random Forest | 1.0 ± 0.0 | 1.0 ± 0.0 | 1.0 ± 0.0 | 1.0 ± 0.0 |
|  | SVM (RBF Kernel) | 0.781 ± 0.033 | 0.671 ± 0.047 | 0.792 ± 0.027 | 0.813 ± 0.028 |
|  | Linear SVM (Heuristic C) | 0.822 ± 0.002 | 0.722 ± 0.004 | 0.802 ± 0.002 | 0.864 ± 0.003 |
|  | Logit (Heuristic C) | 0.666 ± 0.003 | 0.584 ± 0.003 | 0.738 ± 0.002 | 0.728 ± 0.003 |
|  | Stacked | 0.736 ± 0.005 | 0.604 ± 0.006 | 0.77 ± 0.002 | 0.762 ± 0.004 |
| Sleep duration | Extra Trees | 1.0 ± 0.0 | 1.0 ± 0.0 | 1.0 ± 0.0 | 1.0 ± 0.0 |
|  | Linear SVM | 0.816 ± 0.002 | 0.725 ± 0.003 | 0.799 ± 0.002 | 0.859 ± 0.002 |
|  | Random Forest | 1.0 ± 0.0 | 1.0 ± 0.0 | 1.0 ± 0.0 | 1.0 ± 0.0 |
|  | SVM (RBF Kernel) | 0.901 ± 0.107 | 0.834 ± 0.176 | 0.898 ± 0.105 | 0.913 ± 0.092 |
|  | Linear SVM (Heuristic C) | 0.818 ± 0.003 | 0.717 ± 0.004 | 0.805 ± 0.002 | 0.86 ± 0.002 |
|  | Logit (Heuristic C) | 0.661 ± 0.004 | 0.572 ± 0.004 | 0.749 ± 0.001 | 0.726 ± 0.004 |
|  | Stacked | 0.736 ± 0.005 | 0.59 ± 0.013 | 0.772 ± 0.004 | 0.763 ± 0.004 |
| Snoring | Extra Trees | 1.0 ± 0.0 | 1.0 ± 0.0 | 1.0 ± 0.0 | 1.0 ± 0.0 |
|  | Linear SVM | 0.786 ± 0.002 | 0.684 ± 0.003 | 0.575 ± 0.005 | 0.682 ± 0.003 |
|  | Random Forest | 1.0 ± 0.0 | 1.0 ± 0.0 | 1.0 ± 0.0 | 1.0 ± 0.0 |
|  | SVM (RBF Kernel) | 0.884 ± 0.094 | 0.822 ± 0.136 | 0.766 ± 0.182 | 0.835 ± 0.134 |
|  | Linear SVM (Heuristic C) | 0.788 ± 0.002 | 0.671 ± 0.003 | 0.545 ± 0.006 | 0.686 ± 0.004 |
|  | Logit (Heuristic C) | 0.681 ± 0.002 | 0.561 ± 0.003 | 0.281 ± 0.01 | 0.539 ± 0.003 |
|  | Stacked | 0.675 ± 0.004 | 0.548 ± 0.006 | 0.205 ± 0.023 | 0.535 ± 0.007 |

Supplementary Table 5: Results of the in-sample evaluation (CV) of the 7 models for each SH-related characteristic. Values represent the mean and standard deviation across CV repetitions.

| SH Characteristic | Model | Test | p-value | p-value CI |
| --- | --- | --- | --- | --- |
| Daytime sleepiness | Logit (Heuristic C) | partial | 0.0 | [0, 0.003] |
|  | Logit (Heuristic C) | full | 0.0 | [0, 0.003] |
|  | SVM (RBF Kernel) | partial | 0.0 | [0, 0.003] |
|  | SVM (RBF Kernel) | full | 0.0 | [0, 0.003] |
| Morning/Evening chronotype | Linear SVM | partial | 0.754 | [0.726, 0.780] |
|  | Linear SVM | full | 0.0 | [0, 0.003] |
|  | Logit (Heuristic C) | partial | 0.153 | [0.131, 0.176] |
|  | Logit (Heuristic C) | full | 0.0 | [0, 0.003] |
|  | SVM (RBF Kernel) | partial | 0.19 | [0.166, 0.215] |
|  | SVM (RBF Kernel) | full | 0.0 | [0, 0.003] |
| Easiness getting up in the morning | Linear SVM | partial | 0.0 | [0, 0.003] |
|  | Linear SVM | full | 0.0 | [0, 0.003] |
| Daytime nap | Logit (Heuristic C) | partial | 0.0 | [0, 0.003] |
|  | Logit (Heuristic C) | full | 0.0 | [0, 0.003] |
|  | SVM (RBF Kernel) | partial | 0.0 | [0, 0.003] |
|  | SVM (RBF Kernel) | full | 0.001 | [2.5e-5, 0.005] |
| Insomnia | Linear SVM | partial | 0.0 | [0, 0.003] |
|  | Linear SVM | full | 0.0 | [0, 0.003] |
|  | Logit (Heuristic C) | partial | 0.0 | [0, 0.003] |
|  | Logit (Heuristic C) | full | 0.0 | [0, 0.003] |
|  | Stacked | partial | 0.0 | [0, 0.003] |
|  | Stacked | full | 0.0 | [0, 0.003] |
| Sleep duration | Linear SVM | partial | 0.0 | [0, 0.003] |
|  | Linear SVM | full | 0.0 | [0, 0.003] |
|  | Logit (Heuristic C) | partial | 0.0 | [0, 0.003] |
|  | Logit (Heuristic C) | full | 0.0 | [0, 0.003] |
|  | Extra Trees | partial | 0.0 | [0, 0.003] |
|  | Extra Trees | full | 0.0 | [0, 0.003] |
| Snoring | Linear SVM | partial | 0.0 | [0, 0.003] |
|  | Linear SVM | full | 0.0 | [0, 0.003] |
|  | Logit (Heuristic C) | partial | 0.0 | [0, 0.003] |
|  | Logit (Heuristic C) | full | 0.0 | [0, 0.003] |

Supplementary Table 6: Results of the confounding bias partial and full tests for the models that were selected as best performing at least for one of the 4 evaluated metrics.

| **SH Characteristic** | **Group** | **N** | **Mean ± SD** | **Stat (t)** | **p-val** | **Effect size (d)** |
| --- | --- | --- | --- | --- | --- | --- |
| Insomnia | Total | 14973 | 63.99 ± 7.60 | 5.39 | 7.35e-08 | 0.09 |
|  | Positive | 8846 | 64.27 ± 7.46 |  |  |  |
|  | Negative | 6127 | 63.59 ± 7.79 |  |  |  |
| Sleep duration | Total | 16719 | 64.39 ± 7.51 | 13.13 | 3.84e-39 | 0.21 |
|  | Positive | 9959 | 65.02 ± 7.36 |  |  |  |
|  | Negative | 6760 | 63.46 ± 7.65 |  |  |  |
| Getting up in the morning | Total | 14241 | 64.51 ± 7.50 | -28.98 | 2.02e-172 | -0.58 |
|  | Positive | 3554 | 61.33 ± 7.70 |  |  |  |
|  | Negative | 10687 | 65.57 ± 7.13 |  |  |  |
| Morning/Evening chronotype | Total | 9543 | 64.05 ± 7.55 | -8.36 | 8.24e-17 | -0.20 |
|  | Positive | 2398 | 62.93 ± 7.66 |  |  |  |
|  | Negative | 7145 | 64.43 ± 7.48 |  |  |  |
| Daytime nap | Total | 17480 | 63.54 ± 7.50 | 21.12 | 5.05e-89 | 0.56 |
|  | Positive | 1565 | 67.32 ± 7.43 |  |  |  |
|  | Negative | 15915 | 63.17 ± 7.41 |  |  |  |
| Snoring | Total | 25908 | 63.98 ± 7.53 | -5.56 | 2.67e-08 | -0.07 |
|  | Positive | 9469 | 63.64 ± 7.25 |  |  |  |
|  | Negative | 16439 | 64.17 ± 7.68 |  |  |  |
| Daytime sleepiness | Total | 27864 | 64.08 ± 7.54 | 20.94 | 1.94e-95 | 0.30 |
|  | Positive | 6458 | 65.79 ± 7.50 |  |  |  |
|  | Negative | 21406 | 63.56 ± 7.47 |  |  |  |

Supplementary Table 7. Summary of Age by group including statistics across extreme responses to each SH characteristic.

| **SH Characteristic** | **Total (M/F)** | **Positive (M/F)** | **Negative (M/F)** | **Stat (χ²)** | **p-val** | **Effect size (φ)** |
| --- | --- | --- | --- | --- | --- | --- |
| Insomnia | 0.97 | 0.70 | 1.58 | 581.14 | 2.12e-128 | 0.20 |
| Sleep duration | 0.90 | 0.98 | 0.79 | 47.50 | 5.51e-12 | 0.05 |
| Getting up in the morning | 0.95 | 0.49 | 1.18 | 471.33 | 1.64e-104 | 0.18 |
| Morning/Evening chronotype | 0.80 | 0.84 | 0.79 | 1.50 | 2.21e-01 | 0.01 |
| Daytime nap | 0.76 | 2.27 | 0.69 | 475.70 | 1.84e-105 | 0.16 |
| Snoring | 0.93 | 1.43 | 0.72 | 699.36 | 4.12e-154 | 0.16 |
| Daytime sleepiness | 0.89 | 1.13 | 0.83 | 123.68 | 9.91e-29 | 0.07 |

Supplementary Table 8. Summary of Sex ratio (male/female) by group including statistics across extreme responses to each SH characteristic.

Supplementary Figure 1: Side-by-side comparison of the best model for each SH-related characteristic using either brain features (blue), age and sex (orange) and all non-imaging-derived confounds (green), for both threshold-dependent and threshold-independent metrics. Each panel depicts the performance using a different metric: A) Area under the receiver-operator characteristic (ROC-AUC), B) Balanced Accuracy, C) F1-Score, D) Average precision. Each dot represents the performance obtained at each of the 25 test folds within cross-validation (CV). Boxplots summarize the medians and 95% CI for the underlying distribution.

## Reference

1. R: Fast Heuristics For The Estimation Of the C Constant Of A... https://search.r-project.org/CRAN/refmans/LiblineaR/html/heuristicC.html.
